# The Indicator of Emotional Eating and Its Effects on Dietary Patterns among Female Students at Qassim University

**DOI:** 10.3390/nu15163553

**Published:** 2023-08-11

**Authors:** Razan M. Alharbi, Hend F. Alharbi

**Affiliations:** Department of Food Science and Human Nutrition, College of Agriculture and Veterinary Medicine, Qassim University, Buraydah 51452, Saudi Arabia; 411200231@qu.edu.sa

**Keywords:** emotional eating, dietary patterns, food frequency questionnaire, students, university, nutrition

## Abstract

Emotional eating (EE) is considered as the inclination to eat in response to emotions and is associated with certain syndromes. In this sense, we explored the indices of EE and its association with dietary patterns among female students at Qassim University in Saudi Arabia. A cross-sectional study of 380 participants (aged 18–29 years, female students) was performed. Anthropometric measurements of the participants were taken, such as height, body mass index, fat mass and hip–waist circumference as well as the Emotional Eating Scale (EES). The classification of the ESS included the thresholds of 44.7, 43.9 and 11.3% for low, moderate and high EE, respectively. Linear regression after adjustment showed that fat intake was a significant predictor of EE (*p* = 0.031) as well as feelings of enthusiasm. We also observed an interesting indication: fat intake (*p* = 0.011) and educational level (*p* < 0.05) were significantly associated with, and could be significant predictors of, EE. The results highlight the importance of emotional eating, its relationship with the consumption of foods that contain fat and understanding how it develops by raising awareness of the importance of healthy food for a healthy lifestyle.

## 1. Introduction

Emotional eating (EE) is an eating disorder that involves increased eating in response to negative emotions. However, EE was initially defined as eating only in response to negative emotions [1]. Several studies have shown that a positive mood can also lead to an increase in food intake, which is also a reaction towards food cravings in response to stress or negative emotions (such as anxiety, anger, depression and loneliness) as an attempt to alleviate these concerning states [2]. Food products can be energy-dense and high in sugars and fats [3]. Some studies have shown a link between EE and the excessive consumption of energy-dense and fat-rich foods [4]. It has also been linked to the consumption of energy and macronutrients, especially fats and carbohydrates [5]. Studies have shown that negative emotions often lead to the excessive consumption of foods rich in fats and sugars, in contrast to the lack of consumption of healthy foods; therefore, affected individuals do not adhere to healthy eating behaviours, such as eating vegetables and fruits, and exhibit negative behaviours, i.e., not eating breakfast, skipping daily meals and increasing calories [6]. The excessive consumption of foods that are high in fat or sugar can lead to weight gain in the long term, which leads to an increase in body mass index and many health risks, such as oxidative stress, inflammation and obesity. Since this eating behaviour involves the consumption of high-calorie foods, it may have a direct effect on weight status [7]. However, EE has been observed in both normal-weight and overweight or obese individuals [8]. It is difficult to predict the effect of emotions on individuals regarding food cravings during the transitional period from adolescence to young adulthood [9]. The transition to university is critical for young people. It is their first test in making their own food choices, and poor cooking skills and obesity-promoting environments with a wide availability of fast food may determine the adoption of unhealthy eating habits and sedentary patterns leading to weight gain, which may persist into adulthood [10]. University students differ from students of other educational stages, with direct or indirect effects on their eating patterns and behaviours. Unhealthy habits are common among college students during the academic year [11,12], including low physical activity, high consumption of junk food, eating snacks in the evening and high levels of perceived stress.

For this reason, these individuals constitute an important target group among whom to promote healthy lifestyles and reduce the risk of chronic non-communicable diseases in adulthood. While university students also show higher anxiety levels than the general population, stress, anxiety and depression are among the psychological problems that occur during university studies [13]. According to a study by Almogbel et al. [14], 28.2% of undergraduate students at Qassim University experienced some level of stress. One study of Spanish university students noted that approximately 44.7% showed emotional distress indicative of anxiety, regardless of their field of study [15]. In a study that assessed the frequency of EE among 335 female university students over 28 days, it was found that half of them reported experiencing these episodes at some time; further, among 51.3% of them, EE was associated with anxiety [16]. Students who experience high levels of stress usually consume more energy-dense foods and foods subjected to extensive cooking and are less likely to exercise, which can lead to obesity and metabolic disorders [17]. Studies indicated that among university students, EE was not related to eating large amounts of foods with high energy levels, carbohydrates and fats [18]; therefore, it is difficult to predict the effects of emotions on eating styles and habits. This is due to the multitude of influences that differ from one individual to another. Sadness can make an individual lose their appetite for food, while boredom increases appetite [19]. A study conducted on University of Bahrain students showed that emotional eating resulted from the students’ responses to the negative feelings they experienced and the psychological pressure they were under [20].

On the other hand, we can observe that students in their first year of university gain excess weight due to EE and the failure of university students to adapt to their new environment following the transition from high school [21,22]. Stress associated with this experience can lead to negative eating habits, such as the excessive consumption of alcohol and anxiety associated with overeating [23,24]. In this study, EE was evaluated using the method of Arnow et al. [25], a tool commonly used in studies of emotion and eating. Therefore, this study aims to demonstrate the prevalence of EE, its effects on dietary patterns and to describe the changes in anthropometrics measurements among female students at Qassim University. To the best of our knowledge, with the lack of results on EE, especially in Saudi Arabia, this study expands the data on EE, its prevalence and its general effect on dietary habits among female students.

## 2. Materials and Methods

### 2.1. Study Design and Participants

This cross-sectional study was conducted among healthy Saudi female students at Qassim University. The data used were collected from four hundred eighty students from several colleges on the main campus of Qassim University. The selection criteria were the following: female sex, age of 18–29 years, residency in Saudi Arabia and status as a Saudi student at Qassim University during the period of study. The exclusion criteria were as follows: non-Saudi citizens; pregnant or lactating women; and those previously diagnosed with sleep and/or psychiatric disorders, gastrointestinal disorders, significant proteinuria or amyloidosis, arthritis, anaemia, malabsorption or comorbid chronic diseases (e.g., thyroid disorders, diabetes mellitus, malignancies and chronic obstructive pulmonary disease. One hundred and two female students were excluded based on the exclusion criteria or due to incomplete or random participation. The participants were asked if they wished to participate in the study, and all 380 female students showed interest in volunteering to participate.

### 2.2. Ethical Consideration

The study was conducted following the Declaration of Helsinki and approved by the Institutional Review Board (or Ethics Committee) of Qassim University (No. 21-03-05, issued on 18 October 2021). Before completing the survey, the participants read and provided informed consent.

### 2.3. Data Collection

The data were collected in a nutritional counselling room at the Department of Food Science and Human Nutrition of Qassim University. The researcher interviewed each female student, explained the questionnaire’s content, answered any questions and took physical measurements. Each female student was informed of the possibility of withdrawing from participation at any time.

### 2.4. Questionnaire

The survey comprises five sections: (1) the survey aims and consent form, (2) demographic information, (3) anthropometric measurements, (4) the Food Frequency Questionnaire and (5) the Emotional Eating Scale (EES).

#### 2.4.1. Demographic Information

The demographic data included age, marital status, number of family members, affiliated university department, education level, grade point average (GPA), income, number of children and area of residence for all participants.

#### 2.4.2. Anthropometrics Measurements

The participants’ body height was measured using a stadiometer at the start of the study. Each time, a tape measure and a waist–hip ratio (WHR) calculator were used to determine the subjects’ waist and hip circumference. All of the measurements were taken and calculated. Obesity types were defined using the WHR index and then classified according to the report in [26].

The students’ body composition was measured using a body composition analyser (Tanita TBF-410GS, Arlington Heights, IL 60005, USA) and the bioelectrical impedance analysis method. The subjects’ body mass index (BMI), Basal Metabolic Rate (BMR), body fat percentage and fat-free mass were calculated according to the methodology described in [27,28].

#### 2.4.3. Emotional Eating Scale (EES)

The validated Arabic version of the Emotional Eating Scale (EES) was used in this study [29]. The EES has been validated based on clinical [25] and non-clinical [30] samples. Participants rate their responses on a five-point Likert scale ranging from 0 (no desire to eat) to 4 (strong desire to eat; an overwhelming urge to eat). The total score is calculated by adding the scores of all the items and can range between 0 and 100 [25]. Scores of 27.5 indicate low EE, scores ranging from 27.6 to 43.6 indicate moderate EE and scores of 43.7 indicate high EE [25,31]. Higher scores indicate a reliance on food to manage emotions.

#### 2.4.4. Food Frequency Questionnaire (FFQ)

The Arabic language questionnaire contains 133 food-related questions. The frequency of eating or the desire to consume sweets (Likert-type scale (hereafter, “Likert scale”)), the desire to consume coffee and tea (Likert scale) and the amount of water consumed are also accounted for. A food frequency questionnaire was also administered to assess macronutrient intake, which included energy intake (kcal/day), fat intake (g/day), carbohydrate intake (g/day) and protein intake (g/day) [32]. The Saudi Food and Drug Authority validated the FFQ, and the responses were analysed using a Microsoft Excel spreadsheet provided by Dr. Majed Alkhalaf [32].

### 2.5. Statistical Analysis

SPSS version 23.0 was used to analyse the data entered into Excel (IBM, Armonk, New York, NY, USA). The mean, percentage differences, *t*-test, linear regression and chi-square test were calculated using an independent sample. The Pearson correlation test was used to calculate correlations between continuous variables. Statistical significance was defined as a *p*-value of less than 0.05.

## 3. Results

### 3.1. The Associations between the Demographics and EE

Table 1 shows the demographic information and its correlations with EE. No significant correlation was identified between moderate EE (18–22) and low EE (23–25). Moreover, there is an equal association of low and moderate EE with unmarried status. As for the level of education at the undergraduate level, there is a convergence between emotional and average eating. The chi-square test showed a significant correlation between the undergraduate level and increased EE, with a significant difference (*p* = 0.03). Likewise, in terms of the academic year, moderate EE is evident in the second and fourth years, with a decrease in EE in the first and fifth years. According to the EE classification of the female students, with a cumulative average of 4.5–5, these students had moderate EE, with a decrease of 3.5–4.5.

**Figure 1:**
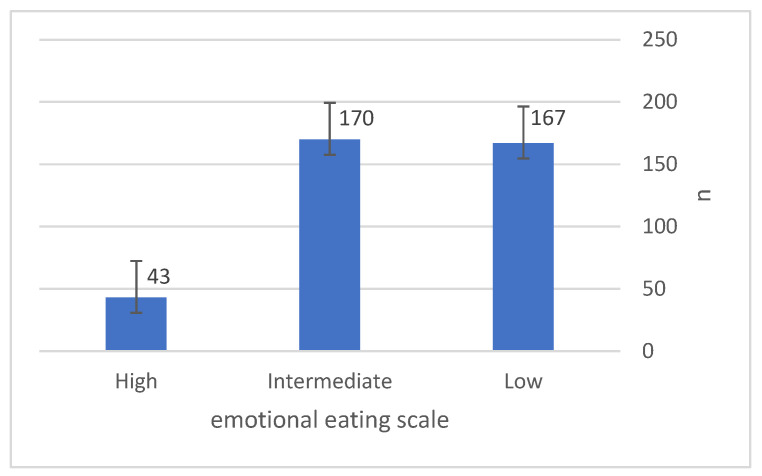
Classification of emotional eating scale (EES).

Figure 1 shows that the mean value of EE is 29.4 ± 12.3. The results of EE classification were very close between low and moderate EE, with values of 170 for low EE (44.7%), 167 for moderate EE (43.9%) and 43 for high EE (11.3%), respectively.

### 3.2. The Association between Eating Patterns and EE

Table 2 shows the eating patterns and habits of the participants. Most participants did not follow a healthy diet at home (187, 49.2%). In fact, nearly half of the participants did not follow a diet. According to the classification of EE, the category of low EE had the highest percentage. It was noted that more than half of the participants, or 198 (52.1%), ate two meals during the day, and dinner was the main meal for 141 (37.1%) of the participants.

When classifying according to EE, the average EE category was higher than the rest, without significant variations. A comparison of the EE index with the main meal of the day showed that for these participants, their main meal was breakfast (*p* = 0.043). They sometimes ate sugary foods, such as biscuits, cookies, etc., and binge drinking was only observed in 196 (51.6%). In contrast, binge drinking of coffee was prevalent, in 224 individuals (58.9%), and the results showed a high demand for tea and coffee; still, the craving for coffee was prevalent.

In contrast, the craving for tea was rare. The category of low EE is higher than the other categories if gluttony is defined as frequently eating sugary foods, and the average EE category was observed to increase when binge drinking of tea was rare. The chi-square test showed no significant difference for the binge drinking of coffee, and 220 (57.9%) people did not include fast food in their daily diet.

As for the participants who ate fast food weekly, 175 (46.1%) ate it once a week; thus, the prevalence of EE was decreased compared with the rest of the groups. In total, 282 (74.2%) participants confirmed that they drank water daily. When classifying EE, more than half of the participants, or 201 (52.9%), had a daily water intake of 1–1.5 litres, and moderate EE was the highest category, with no significant differences.

#### Mean Macronutrient Intake

**Figure 2:**
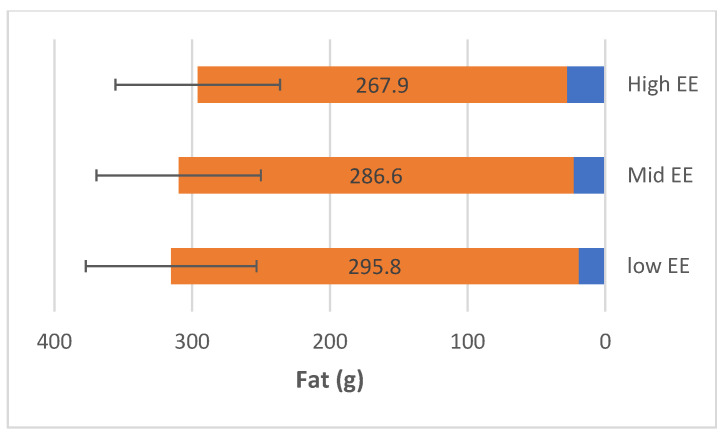
The range of fat consumption (g/day) among the participants.

**Figure 3:**
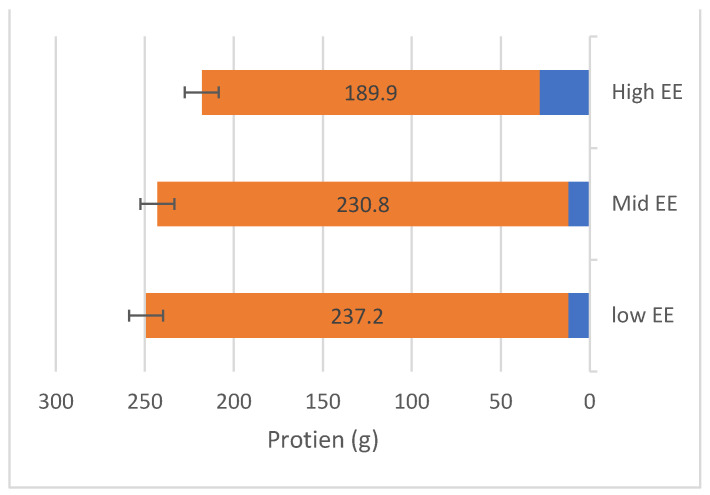
The range of protein consumption (g/day) among the participants.

**Figure 4:**
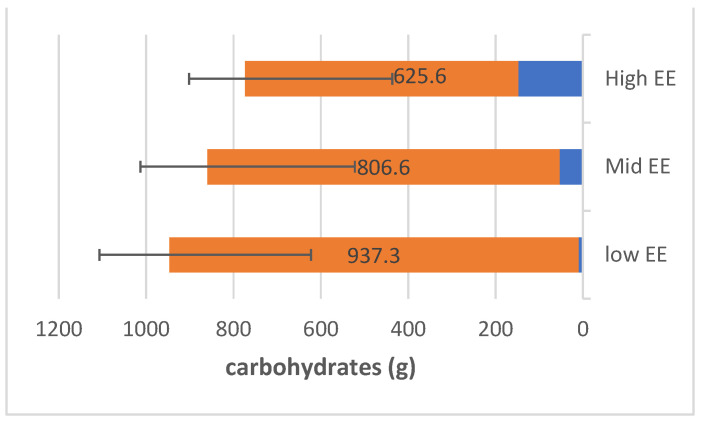
The range of carbohydrate consumption (g/day) among the participants.

FFQ has analysed the mean of intaking the micronutrient. Figure 2 shows participants with higher EE consumed fat from 28 g/day to 267.9 g/day. The range of protein consumption was 189.9 g/day, as shown in Figure 3. The range of carbohydrates of participants with higher EE consumed was 148.2 g/day to 625.6 g/day (Figure 4).

### 3.3. The Association between Anthropometric Measurements and Emotional Eating

As shown in Table 3 the category of low EE constituted the highest category, at 56%, and a decrease in EE was observed in the context of obesity. The average measurement of the percentage of fat for the participants was 24.6 ± 9.4, with a decrease in EE according to the EE classification. The average for the fat-free mass was 42 ± 5.2. According to this classification, the EE category increased. During the measurement of the ratio between waist and hip circumference with respect to the possibility of infection, it was found that 355 (93.4%) of the participants had a low-risk probability. The low EE percentage was higher than that in the remaining categories when they were classified according to EE.

### 3.4. The Linear Regression Analyses

The linear regression analysis, the results of which are shown in the unmodified Table 4, showed that fat intake was the only predictor of an increase in EE (*p* = 0.025). Also, linear regression after adjustment showed that fat intake was a significant predictor of EE, *p* = 0.031. The increase in fat intake significantly predicted the increase in EE by 3.2. As is well-known and can be expected, the increase in fat intake is one of the most vital indicators of EE. Thus, after adjustment, the linear regression analysis results showed that fat intake predicts the increase in EE and the linear regression. The average fat intake was an important predictor of EE.

Interestingly, the linear regression after adjustment showed that educational level is a significant predictor of EE (*p* = 0.011); an increase in educational level significantly predicted an increase in EE, with a coefficient of beta = 5.4.

## 4. Discussion

It was noted during this research on students at Qassim University that most of the respondents lived with their families [33]. According to the reported numbers of family members and servants at home, the number of family members ranged from 8 to 10. The General Authority for Statistics reported in 2019 that the average number of family members was 6.37 [23]. In the present study, the participating students who lived with their parents showed better healthy food behaviours than those who lived far away.

Our results are consistent with previous studies showing that low EE predominates among students living with family, an environment that reduces the effects of EE and replaces it with different activities [34]. Considering the relationship between the number of family members and EE, we did not find any statistical evidence to support this [35]. The city population was also associated with decreased EE, as were female participants living in Buraidah.

A similar study was conducted on women in Riyadh [36]. The average EE was 27.5 ± 16. The study reported that 40.4% of participants had low EE and 12.4% had high EE [31]. Moreover, in Turkey, a study was conducted at Kahramanmaraş University, Suzhou Imam, to determine the effects of the social and demographic characteristics of 537 university students from health colleges on their emotions and eating behaviours. The average EE among the students was 73.29 ± 20.85, which falls within the average EE range according to the scale employed here [37]. This finding contradicted the study reported in [35], whose authors stated that 38.6% of the study sample were emotional eaters. This difference is due to the tools used to assess EE efficacy. The ascertained baseline compositions may not be directly comparable, and the difference may also be due to geographic distance.

Similarly, a study conducted at the University of Sharjah showed that 45.3% of students did not follow a diet [38] due to the academic life of university students and their busy exam schedules. Some students tend to avoid prioritizing their health and the quality of their food during the day. The reason for this is that skipping healthy meals at the beginning of the day and consuming coffee, sandwiches and croissants high in sugar and calories can affect sugar levels, making one’s mood sour for the rest of the day. After extensive searching through the literature, we did not find reports that agreed with the present research results on the association of eating breakfast with high EE. At the same time, some studies contradicted the notion that skipping breakfast can improve mood or that skipping a meal can have bad behavioural and mood effects. Despite the fact that our participants ate breakfast, the findings indicate that adolescents who skipped breakfast had better HRQOL (health-related quality of life), lower stress and less depression than those who ate this meal [39]. Commercial baked goods characterise poor-quality and low-nutrient breakfasts, while it is agreed that skipping breakfast potentially affects health behaviours. Risk factors and poor mental indicators also play a role [40,41,42]. One study found an association between skipping breakfast and depression. The results showed that participants who ate breakfast rarely or sometimes were more likely to have depressive symptoms than those who ate it frequently [43]. This contradiction is due to differences in research aims, the features of participants, locations of study, tools and age.

These results reflect the social view that coffee holds a prominent place as part of Gulf countries’ hospitality values. There are similar results from the University of Bahrain and the United Arab Emirates, where students consumed large amounts of coffee throughout the day due to the customs and traditions in the Gulf regarding coffee consumption [44,45]. The recent results indicated that females consume more coffee than males, which was explained by female students’ lifestyles and resting places as compared to those of males in the Arab world when using EE classifications.

The prevalence of obesity in Saudi Arabia is the highest in the world [46]; in 2019, the Saudi Ministry of Health released the latest data from the World Health Organization’s Global Health Survey (KSAWHS). The data showed that 20% of people in the Kingdom of Saudi Arabia have obesity, with a higher percentage among females (21%) [47], and 63 (16.6%) are underweight (29.5%), overweight (20.8%) or obese (8.7%). These numbers are much lower than the recent national average of 38% reported for young Saudis who are overweight and obese [48]. These results are similar to the results of recent studies conducted in Saudi Arabia on female university students [49,50,51,52]. One study compared overweight and obesity in terms of prevalence [53]. The results were much lower, with 13.95% and 11.63% for overweight and obesity, respectively. It is important to consider regional differences and the places of residence of people, as the existence of cultural differences may affect people’s food habits and lifestyle.

This study found no significant relationship between EE and BMI [31]. Korean research also indicated that EE was not associated with physical measurements related to obesity, such as BMI, fat mass or waist–hip circumference [54]. EE was not associated with body mass index in a sample of overweight individuals [55]. Other researchers [8,56] believe that negative EE can be a risk factor for weight gain, and one study’s results indicated that a lack of emotional regulation directly affected BMI through EE. Other studies also stated that higher levels of emotional regulation were associated with higher levels of EE, which, in turn, led to higher levels of BMI [57].

These results support the theory that the frequency of cravings for fatty foods is a mediator of the relationship with the tendency to eat in response to emotional feelings. This study’s results agree with [31], where fat intake was a response to EE, while another study indicated that EE was associated with fats and snacks in Riyadh [35]. A study conducted at the University of Bahrain concluded that students consumed high-calorie and high-fat foods during episodes of EE [20]. A study on adolescent students in Taiwan found that EE is associated with the frequent consumption of fast foods rich in fat and unhealthy foods [58]. There is also a correlation between stress rate and excess fat, which may be associated with negative mood [59]. However, this study observed a different trend, proving that EE directly affects fat intake [60]. In contrast to our findings, in a 2019 US study, increased EE was associated with decreased cravings for fatty foods [61].

A study conducted in Turkey showed that a higher level of education leads to an increase in the tendency to eat when facing negative situations, and the researchers suggested that the main reason for this increase in demand for food is the improvement of one’s financial situation with the increase in education [62]. In a study conducted on a sample of Dutch adults to assess nutritional status during COVID-19 quarantine, the participants with a higher level of education reported increased demand for, purchases of, and access to food throughout quarantine. They were eating in response to emotion, compared with the low-educated participants. A high percentage of the former worked at home, while among those with low education, only 9%, worked at home, because low education influenced the nature of their work during the quarantine period. Meanwhile, higher education allowed the former group to work remotely and delivery services facilitated this, which led to an increase in the availability of unhealthy options [63]. Other findings also showed that women with a higher university education were more likely to eat emotionally than women with a more vocational education [64]. In contrast, one study found that employees with a low education level are more likely to develop emotional behaviours in eating compared with employees whose education is high [65]. These discrepancies can be explained by the different goals and designs of various studies.

The current study has many limitations, as it was conducted in a non-clinical setting. Thus, the results cannot be considered representative of the populations of other cities and regions in the Kingdom of Saudi Arabia and other generations. Therefore, studies should be conducted with a larger sample size of participants from different areas in the Kingdom of Saudi Arabia. To improve dietary intake among this young population, future interventions may take into account stress management and eating self-regulation. Another limitation of this study is that it relied solely on what the participants reported, and self-reported measures of EE are subject to bias. Because the study did not use a longitudinal design, it did not follow the participants over an extended period. Thus, behaviour change during different times of the year, such as periods of fasting or exams, could not be observed or measured. Given the study’s greater focus on macronutrient intake through the use of the FFQ, there are several strengths of this study. First, it focused on young women likely to be affected by EE and at increased risk of developing emotional disorders. Secondly, this study included healthy young women representing a group that had not been studied before at Qassim University.

## 5. Conclusions

In conclusion, emotional eating is an important indicator that leads to eating disorders, obesity or a high intake of fat-containing foods, increasing the incidence of metabolic diseases such as diabetes, heart disease and others. Fat intake contributed to and predicted the increase in EE and feelings of enthusiasm. A significant correlation between the undergraduate level and increased EE was identified. Understanding EE may provide more information about the lifestyle of female youth in Saudi Arabia. Moreover, these data could predict certain metabolic syndromes related to changing eating disorders. This research provides further data that could contribute to the creation of a database of young women’s dietary patterns and foods, especially in Saudi Arabia or the Gulf countries. They also stress the importance of EE and its relationship with the consumption of foods that contain fat and of understanding how this develops by raising awareness of the importance of healthy food for a healthy lifestyle.

## Figures and Tables

**Table 1:** The associations between demographic features and EE.

Variables	Item	EES *n* (%)			*p*-Value
		Low	Moderate	High	
Age	18–22	137 (82)	148 (87.1)	35 (81.4)	0.259
	23–25	24 (14.4)	16 (9.4)	4 (9.3)	
	26–29	6 (3.6)	6 (3.5)	4 (9.3)	
Marital status	Single	158 (94.6)	158 (92.9)	42 (97.7)	0.473
	Married	9 (5.4)	12 (7.1)	1 (2.3)	
Education level	Bachelor	162 (97)	163 (95.9)	38 (88.4)	0.023 *
	Residency	0 (0)	0 (0)	1 (2.3)	
	Master	5 (3)	7 (4.1)	4 (9.3)	
GPA	Fresh students	11 (6.6)	15 (8.8)	3 (7)	0.585
	<2.5	1 (0.6)	0 (0)	0 (0)	
	2.5–3.5	15 (9)	21 (12.4)	5 (11.6)	
	3.5–4.5	69 (41.3)	51 (30)	15 (34.9)	
	4.5–5	71 (42.5)	83 (48.8)	20 (46.5)	
Family income level	<5 K SR	20 (12)	19 (11.2)	2 (4.7)	0.767
	5–10 K SR	44 (26.3)	50 (29.4)	16 (37.2)	
	10–15 K SR	48 (28.7)	46 (27.1)	12 (27.9)	
	>15 K SR	55 (32.9)	55 (32.4)	13 (30.2)	
Number of family members	2–4	12 (7.2)	14 (8.2)	3 (7)	0.03 *
	5–7	42 (25.1)	64 (37.6)	20 (46.5)	
	8–10	98 (58.7)	71 (41.8)	17 (39.5)	
	>10	15 (9)	21 (12.4)	3 (7)	

Data presented as *n* (%) for categorical variables; grade point average (GPA); * *p*-value < 0.05 considered significant.

**Table 2:** The associations between eating patterns and EE.

Variables	Items	EES *n* (%)			*p*-Value
		Low	Moderate	High	
Following a healthy eating pattern at home	Yes	38 (22.8)	37 (21.8)	12 (27.9)	0.341
	No	88 (52.7)	77 (45.3)	22 (51.2)	
	Sometimes	41 (24.6)	56 (32.9)	9 (20.9)	
Number of meals/day	One meal	28 (16.8)	21 (12.4)	8 (18.6)	0.846
	Two meals	83 (49.7)	93 (54.7)	22 (51.2)	
	Three meals	47 (28.1)	44 (25.9)	10 (23.3)	
	Four or more meals	9 (5.4)	12 (7.1)	3 (7)	
Main meal/day	Breakfast	42 (25.1)	50 (29.4)	19 (44.2)	0.043 *
	Lunch	54 (32.3)	65 (38.2)	9 (20.9)	
	Dinner	71 (42.5)	55 (32.4)	15 (34.9)	
Having breakfast	Yes	67 (40.1)	80 (47.1)	24 (55.8)	0.409
	No	34 (20.4)	31 (18.2)	6 (14)	
	Sometimes	66 (39.5)	59 (34.7)	13 (30.2)	
Cravings for sugary foods (cookies/chocolate/ice cream, etc.)	Rare	32 (19.2)	28 (16.5)	6 (14)	0.691
	Sometimes	93 (55.7)	91 (53.5)	22 (51.2)	
	Always	42 (25.1)	51 (30)	15 (34.9)	
Craving for tea	Rare	85 (50.9)	90 (52.9)	21 (48.8)	0.990
	Sometimes	53 (31.7)	52 (30.6)	14 (32.6)	
	Always	29 (17.4)	28 (16.5)	8 (18.6)	
Craving for coffee	Rare	24 (14.4)	23 (13.5)	7 (16.3)	0.675
	Sometimes	49 (29.3)	40 (23.5)	13 (30.2)	
	Always	94 (56.3)	107 (62.9)	23 (53.5)	
Fast food consumption/week	Once	82 (49.1)	78 (45.9)	15 (34.9)	0.303
	Twice	28 (16.8)	42 (24.7)	13 (30.2)	
	Three times	20 (12)	15 (8.8)	7 (16.3)	
	Four or more times	3 (1.8)	5 (2.9)	2 (4.7)	
	I do not consume	34 (20.4)	30 (17.6)	6 (14)	
Water consumption/day	<1 L	48 (28.7)	47 (27.6)	15 (34.9)	0.952
	1–1.5 L	89 (53.3)	93 (54.7)	19 (44.2)	
	1.5–2 L	19 (11.4)	19 (11.2)	6 (14)	
	>2 L	11 (6.6)	11 (6.5)	3 (7)	
Supplement intake	Multivitamins	30 (18.4)	33 (17.5)	11 (19)	0.505
	Vitamin D	39 (23.9)	46 (24.3)	14 (24.1)	
	Vitamin B_12_	16 (9.8)	21 (11.1)	4 (6.9)	
	Vitamin C	11 (6.7)	12 (6.3)	7 (12.0)	
	Folic acid	10 (6.1)	12 (6.3)	2 (3.6)	
	Iron	34 (20.8)	24 (12.7)	8 (13.8)	
	Cod liver oil	6 (3.7)	13 (6.8)	3 (5.1)	
	Protein supplements/bars	16 (9.8)	26 (13.8)	8 (13.8)	
	Chlorophyll	0 (0.0)	1 (0.5)	0 (0.0)	
	Zinc	1 (0.6)	1 (0.5)	0 (0.0)	

Data presented as *n* (%) for categorical variables; * *p*-value < 0.05 considered significant.

**Table 3:** The associations between body anthropometrics and EE.

Variables	Items	EES *n* (%)			*p*-Value
		Low	Moderate	High	
BMI	Underweight	29 (17.4)	29 (17.1)	5 (11.6)	0.562
	Normal weight	94 (56.3)	90 (52.9)	21 (48.8)	
	Overweight	28 (16.8)	38 (22.4)	13 (30.2)	
	Obese	16 (9.6)	13 (7.6)	4 (9.3)	
	Range	2.8–48.4	1.3–46.1	2.8–48.6	
	Mean ± SD	24.6 ± 9.4	23.6 ± 9.3	23.6 ± 10.8	
FFM	Range	27–60.8	9.7–58	34.1–58.8	0.521
	Mean ± SD	41.9 ± 4.7	41.9 ± 5.6	42.9 ± 5.5	
WHR	Low	159 (95.2)	158 (92.9)	38 (88.4)	0.299
	Intermediate	7 (4.2)	7 (4.1)	4 (9.3)	
	High	1 (0.6)	5 (2.9)	1 (2.3)	

Data presented as the mean ± SD, *n* (%). *p*-value < 0.05 is considered significant.

**Table 4:** The associations between the demographic features and EE using linear regression analyses.

Variables	Unadjusted			Adjusted			Adj. R^2^
	B ± SE	Std. Beta	*p*-Value	B ± SE	Std. Beta	*p*-Value	
Age	0.006 ± 1.277	0.000	0.996	−3.081 ± 1.833	−0.124	0.094	0.007
Education level	2.999 ± 1.553	0.099	0.054	5.403 ± 2.126	0.178	0.011 *	0.017
Family income level	0.178 ± 0.627	0.015	0.777	0.206 ± 0.642	0.017	0.749	0.000
BMI	0.004 ± 0.132	0.002	0.975	0.045 ± 0.133	0.018	0.735	0.000
Daily activity	0.507 ± 0.948	0.027	0.593	0.653 ± 0.965	0.035	0.499	0.001
Number of meals	−0.393 ± 0.808	−0.025	0.627	−0.287 ± 0.82	−0.018	0.726	0.000
Fat intake	2.978 ± 1.322	0.115	0.025 *	3.243 ± 1.496	0.125	0.031 *	0.012
Sugar intake	0.863 ± 1.496	0.030	0.564	−0.678 ± 1.659	−0.023	0.683	0.000
Carb intake	−2.943 ± 7.130	−0.021	0.680	−1.492 ± 7.176	−0.011	0.835	0.000

Data presented as BSE from linear regression. *p*-value adjusted for age, BMI, income and diet. * *p* < 0.05 was considered statistically significant.

## Data Availability

Data is contained within the article.
